# Supplementary material for: Disability and depression among stroke survivors attending rehabilitation facilities at three designated tertiary care hospitals in Bangladesh: A cross-sectional study
Source: PLoS One. 2025 Jan 3;20(1):e0311325. doi: 10.1371/journal.pone.0311325 (PMC11698461; doi:10.1371/journal.pone.0311325)
Supplement: Attachment — Submitted filename: jeviwer_j-stroke-1.docx [file pone.0311325.s003.docx]

STROBE Statement—Checklist of items that should be included in reports of ***cohort studies***

|  | Item No | Recommendation | Page No |
| --- | --- | --- | --- |
| **Title and abstract** |  | Title is clear and addressed an important issue of health significance  Abstract is structured and presented the findings though the world limit exceeded 300. | |
| Introduction | | | |
| Background/rationale |  | The scientific background and rationale is well addressed  Clear and concise | |
| Objectives |  |  |  |
| Methods | | | |
|  |  | The design, settings, periods and participants are well described  The eligibility criteria are properly addressed  Outcome variables are described and used appropriate tool for data collection. The Statistical methods employed is sufficient  Nevertheless, Authors need to describe how data quality was assured (mention pretesting, standardization of data collection method, was training given etc… | |
| Results | | | |
| Participants |  | Surprising to see 100.0% response rate? How this was possible given the study is facility based and sometimes we see missing information? Elaborate on this further to ensure the credibility of the work.  Report numbers of individuals including the percentages and use appropriate scientific language | |
| Discussion | | | |
|  |  | The key findings are summarized with reference to study objectives  The strength and limitations of the study is well described, taking into account sources of potential bias or imprecision. | |
|  |  |  |  |
| Other information |  | Authors need to acknowledge all who assisted/participated in tins study  Mention the contribution of authors  Interestingly, authors have attached the raw data | |
|  | | |  |

Overall impression: the paper is suitable for publication with minor editorial and language improvemets.
