# Supplementary material for: Disability and depression among stroke survivors attending rehabilitation facilities at three designated tertiary care hospitals in Bangladesh: A cross-sectional study
Source: PLoS One. 2025 Jan 3;20(1):e0311325. doi: 10.1371/journal.pone.0311325 (PMC11698461; doi:10.1371/journal.pone.0311325)
Supplement: Attachment — Submitted filename: Response to Reviewers.docx [file pone.0311325.s004.docx]

***Manuscript Title:*** ***Disability and depression among stroke survivors attending rehabilitation facilities at three designated tertiary care hospitals in Bangladesh: A cross-sectional study***

**Reply to the reviewer’s comments**

We express our gratitude to the reviewer for their meticulous and in-depth reading of this manuscript, as well as for their insightful remarks and helpful recommendations, all of which have enabled us to enhance the text's quality.

**Reviewer # 3**

We express our gratitude to the reviewer for their meticulous and comprehensive evaluation of this paper, as well as for their insightful remarks and valuable recommendations, which significantly contributed to enhancing the overall quality of this work.

**Title and Abstract**

**Comment No: 1** Title is clear and addressed an important issue of health significance. Abstract is structured and presented the findings though the world limit exceeded 300.

**Reply No: 1** Thank you for your insightful feedback. In compliance with the journal guidelines, we have decreased the word count to 300.

**Introduction**

**Comment No: 2** The scientific background and rationale is well addressed. Objectives Clear and concise

**Reply No: 2** Thank you for your valuable comment

**Methodology**

**Comment No: 3**

The design, settings, periods and participants are well described

The eligibility criteria are properly addressed

Outcome variables are described and used appropriate tool for data collection. The Statistical methods employed is sufficient

Nevertheless, Authors need to describe how data quality was assured (mention pretesting, standardization of data collection method, was training given etc…

**Reply No: 3** Thank you for your positive feedback. The following statements has been added in the revised text: The interviews were performed by three proficient data collectors, all of whom were physiotherapy graduates with more than three years of experience. Prior to data collection, a training session was conducted to demonstrate data collection process and maintain consistency among the data collectors. In order to address any issues that may arise during data collection, each data collector carried out a pre-test session including a minimum of five data. These data were excluded from the final analysis.

**Results**

**Comment No: 4** Surprising to see 100.0% response rate? How this was possible given the study is facility based and sometimes we see missing information? Elaborate on this further to ensure the credibility of the work.

Report numbers of individuals including the percentages and use appropriate scientific language

**Reply No: 4** We appreciate you posing this inquiry and affording us the chance to provide reasonable justifications. Our data collection process utilized the echo-friendly Google Forms platform as an alternative to traditional pen and paper methods. The inclusion of the mandatory option on each question serves to avoid incomplete submissions.

**Discussion**

**Comment No: 5** The key findings are summarized with reference to study objectives

The strength and limitations of the study is well described, taking into account sources of potential bias or imprecision.

**Reply No: 5** Thank you for your positive feedback.

**Other information**

**Comment No: 6** Authors need to acknowledge all who assisted/participated in tins study

**Reply No: 6** That material has already been provided in the acknowledgement section.

**Comment No: 7** Mention the contribution of authors

**Reply No: 7** Your insightful remark is appreciated. The information has already been incorporated into the journal system in accordance with the prescribed guidelines of the journal.

**Comment No: 8** Interestingly, authors have attached the raw data

**Reply No: 8** Raw data was included as per journals requirement
